# Teleconsultation use and satisfaction among cancerologists during the COVID-19 pandemic in Morocco

**DOI:** 10.11604/pamj.2023.44.89.35081

**Published:** 2023-02-16

**Authors:** Bouchra Amaoui, Laila Lahlou, Fatima Safini, Slimane Semghouli

**Affiliations:** 1 Faculty of Medicine and Pharmacy, University Ibn Zohr, Agadir, Morocco; 2 Regional Center of Oncology, Agadir, Morocco; 3 Higher Institute of Nursing Professions and Health Techniques, Agadir, Morocco

**Keywords:** Teleconsultation, COVID-19, cancerologists, perception, satisfaction, Morocco

## Abstract

**Introduction:**

health care benefits have undergone major changes during the COVID-19 pandemic. This has led to an explosive growth in teleconsultation services mainly for cancer patients. The purpose of this study was to assess the perception and experience of Moroccan oncologists with the use of teleconsultation during the COVID-19 pandemic.

**Methods:**

a 17-question anonymous cross-sectional survey was conducted on Google forms and emailed to all Moroccan oncologists. Statistical analysis was performed using the statistical software Jamovi (version 2.2).

**Results:**

out of a total of 500 oncologists who received the questionnaire, 126 responded, with a response rate of 25%. During the pandemic, only 59.5% of oncologists used teleconsultation, with no significant differences among the three groups (radiation oncologists, medical oncologists and cancer surgeons (p=0.294)). Most participants were satisfied with being able to explain medical diagnosis, provide assessment results, and provide treatment recommendations during teleconsultation. Finally, 47.2% of participants were willing to continue conducting teleconsultations after the COVID-19 pandemic, with no significant differences among the three groups.

**Conclusion:**

oncology physicians were satisfied with their experiences with teleconsultation and agreed that it is likely to be part of their long-term practice. Future studies are needed to assess patient satisfaction with teleconsultation and to improve patient care through this virtual technology.

## Introduction

The first cases of the coronavirus disease (COVID-19) were detected in the Chinese city Wuhan in December 2019. Then, it quickly spread to most countries, causing a big health crisis and quarantining half of the world's population. The World Health Organization (WHO) qualified it as a pandemic on March 11^th^, 2020 [1]. Centralized global figures released by Johns Hopkins University report on January 6^th^, 2022, at 6:05p.m, 297.8 million confirmed cases of Covid-19 worldwide and 5,465,315 deaths [2]. In Morocco, following the registration of the first case of COVID-19 in the city of Casablanca on March 2^th^, 2020, the disease caused 14,872 deaths and 977,579 cases confirmed until 6^th^ January 2022 at 6:05p.m [2]. Indeed, COVID-19 has impacted all sectors of human activity, which has pushed the authorities of most countries to take measures such as the introduction of social distancing or even confinement. This had an impact on medical practices in most countries [3]. Indeed, the need to mobilize healthcare staff in COVID-19 patient care units, the increase in the number of consultations and the demand for care have exploded. As a result, all healthcare activities have been affected primarily the management of cancer patients who are more likely to manifest a severe or even lethal form of COVID-19 [3-5]. Telemedicine, as a solution to be developed to improve the health care and well-being of patients especially in demedicalized areas, was recommended by the WHO in 2009 [6]. Subsequently, a global eHealth Observatory was established to provide Member States with credible information on the benefits, limitations, and standards of telemedicine [6]. Thus, the first guidelines were published by WHO on 17^th^ April 2019 on digital health interventions [7]. With the COVID-19 pandemic, the European Society of Medical Oncology (ESMO) has recommended teleconsultation as a tool for patient care during this period of health emergency [8].

Indeed, most countries have legislated telemedicine and have encouraged the use of this tool mainly for chronic diseases and cancer patients during the COVID-19 pandemic [9,10]. Thus, the introduction of teleconsultation into routine cancer practices in the USA has been accelerated by a national policy that has regulated its confidentiality and legalized its payment during the COVID-19 pandemic [11]. In addition, a national survey by the American Society of Radiation Oncology (ASTRO) showed that 89% of clinics offer teleconsultation programs to patients [12]. Indeed, teleconsultation has been recommended for the sorting of new patients according to their degree of severity and also for the follow-up of old patients who do not require a clinical examination [13]. It has also been proposed to provide multidisciplinary advice on tumors, educational conferences, breast screening, supportive care, oral chemotherapy treatments, pre-anesthetic consultation and even radiotherapy treatment planning [12,14-16]. In France, the high health authority recommended maintaining the follow-up of cancer patients with their attending physician by teleconsultation from April 2020 [4,17]. It also proposed amendments to allow the use of solutions from the general public (Skype, WhatsApp, Facetime etc.) or the telephone as teleconsultation tools and thus cancel the constraints that only allow audiovisual teleconsultations [18]. In Morocco, the use of telemedicine was initiated by Law No. 131-13 on the practice of medicine in 2015 [19]. The terms of application and reimbursement rules for telemedicine have been regulated by Decree No. 2-18-378 of 25^th^ July 2018 [20]. With the COVID-19 pandemic and in order to minimize the risk of transmission of the coronavirus to patients and service providers, the government has promulgated decrees and laws to relax the use of telemedicine in general and teleconsultation in particular and thus encourage healthcare institutions to use it with greater flexibility [21]. The absence of studies in Morocco on the use of teleconsultation in the medical field and more specifically in the field of oncology, has led us to undertake this work which aims to assess the perception and experience of Moroccan oncologists towards the use of teleconsultation during the COVID-19 pandemic.

## Methods

**Study design and setting**: this was a cross-sectional observational study with a descriptive and analytical purpose. A cross-sectional survey on teleconsultation was carried out among carcinologists, practicing in cancer institutions and members of the Moroccan Society of Cancerology, between September 15^th^ and December 15^th^, 2021.

**Study population**: the study focused on all Moroccan oncologists; Radio-oncologists (RO), Medical Oncologists (MO) and Cancer Surgeons (CS) working in the public and private sectors as well as in the University Hospital Centers (CHU) and who responded to our questionnaire.

**Data collection**: data was collected using a web questionnaire that has been developed on Google Forms. It consisted of a total of 17 questions. The questions were inspired by the American Society for Radiation Oncology's survey of American onco-radiotherapists [11]. The first section of the questionnaire deals with the demographic characteristics of the participants. The second is to collect data on teleconsultation practices. The third section concerns the participants´ perception and satisfaction of teleconsultation. Questions about participants' perception and satisfaction were answered on a 5-point Likert scale ranging from strongly agree, agree, neutral, disagree or strongly disagree with the statement at hand. Responses were confidential, voluntary, and anonymous.

**Statistical analysis**: the statistical analysis was done by the Statistical Software Jamovi (Version 2.2). Qualitative variables were described in numbers and percentages and then compared by Khi2 test or Fisher exact depending on the conditions of application of each of the tests [22,23].

**Ethical considerations**: the study was approved by the Ethical Committee of the Faculty of Medicine and Pharmacy, Ibn Zohr University of Agadir, Morocco. During all stages of this study, the data were only used for the purposes assigned and were considered anonymous and confidential.

## Results

**Demographic characteristics**: out of a total of 500 cancerologists who received the questionnaire, 126 responded, a response rate of 25%. The breakdown by specialty was 42% of ROs, 41% of CSs and 17% of MOs. One in two participants works at a University Hospital Centre (UHC) with no significant difference between the three groups (p=0.083). Table 1 summarizes the demographics of the participants in this survey. The percentage of practitioners over the age of 46 years for ROs, CSs and MOs was 64.3%, 28.6% and 7.1% respectively (p=0.026) (Table 2, Table 2(suite)) presents the survey questions, the percentages of responses by groups and the p-value.

**Table 1:** data demographics of participants

Variable		Radiotherapists	Medical oncologists	Surgeons	Total	P-value
	Observed	53	21	52	126	
**Age range**						
< 25 years	Observed(%)	0(0)	1(4.8)	0(0)	1(0.8)	<0.001
25 to 35 years	Observed(%)	17(32.1)	3(14.3)	34(65.4)	54(42.9)	
35 to 45 years	Observed(%)	18(34.0)	15(71.4)	10(19.2)	43(34.1)	
>45years	Observed(%)	18(34.0)	2(9.5)	8(15.4)	28(22.2)	
**Sectors of activity**						
Private	Observed(%)	16(30.2)	4(19.0)	8(15.4)	28(22.2)	0.083
Public	Observed(%)	12(22.6)	2(9.5)	18(34.6)	32(25.4)	
UHC	Observed(%)	25(47.2)	15(71.4)	26(50.0)	66(52.4)	

**Table 2:** survey questions, number and response rates by specialty groups

Questions			RTs	OMs	SCs	Total	P-value
**During the COVID-19 pandemic,did you resort to teleconsultation?**	Very often	Observed(%)	13 (24.5)	6 (28.6)	7 (13.5)	26 (20.6)	0.516
	Often	Observed(%)	23(43.4)	7(33.3)	19(36.5)	49(38.9)	
	Rarely	Observed(%)	10(18.9)	5 (23.8)	14 (26.9)	29 (23.0)	
	Never	Observed (%)	7 (13.2)	3(14.3)	12(23.1)	(17.5)	
**During the COVID-19 pandemic, please indicate what communication methods has your institution adopted for the conduct of teleconsultations?**	Audio only	Observed (%)	33(63.5)	10 (47.6)	21 (40.4)	64 (51.2)	0.125
	Audio and video	Observed(%)	6 (11.5)	4 (19)	15(28.8)	25(20.0)	
	None	Observed (%)	13 (25)	7 (33.3)	16 (30.8)	36 (28.8)	
**What is the main mode of communication you use for full teleconsultations?**	Audioonly	Observed (%)	20(38.5)	11(57.9)	9(17.6)	40(32.8)	0.003
	Audio and video	Observed (%)	6 (11.5)	6 (31.6)	10 (19.6)	22 (18)	
	WhatsApp	Observed (%)	22(42.3)	2(10.5)	25(49.0)	49 (40.2)	
	SMS	Observed (%)	2 (3.8)	0 (0)	6 (11.8)	8 (6.6)	
	Other	Observed (%)	2 (3.8)	0 (0)	1 (2)	3 (2.5)	
**Prior to the COVID-19 pandemic, did you regularly conduct teleconsultations?**	Yes	Observed (%)	12 (23.1)	13 (61.9)	14 (26.9)	39 (31.2)	0.035
	Rarely	Observed (%)	29 (55.8)	6 (28.6)	26 (50)	61 (48.8)	
	Never	Observed (%)	11 (21.2)	2 (9.5)	12 (23.1)	25 (20)	
**Given the COVID-19 pandemic, do you think it was necessary to move from on-site consultation to remote consultation?**	Totally agree	Observed (%)	18 (34)	9 (42.9)	18 (34.6)	45 (35.7)	0.294
	Agree	Observed %	14 (26.4)	8 (38.1)	16 (30.8)	38 (30.2)	
	Neutral	Observed (%)	9 (17)	3 (14.3)	6 (11.5)	18(14.3)	
	Disagree	Observed (%)	11(20.8)	0(0)	7(13.5)	18(14.3)	
	Strongly disagree	Observed (%)	1(1.9)	1 (4.8)	5 (9.6)	7 (5.6)	
**Do you think that carrying out a complete teleconsultation is impersonal?**	Totally agree	Observed(%)	9(17)	3(14.3)	5(9.6)	17(13.5)	0.323
	Agree	Observed(%)	10(18.9)	8(38.1)	19(36.5)	37 (29.4)	
	Neutral	Observed(%)	17(32.1)	7 (33.3)	20(38.5)	44(34.9)	
	Disagree	Observed (%)	15 (28.3)	3 (14.3)	7(13.5)	25 (19.8)	
	Strongly disagree	Observed (%)	2(3.8)	0(0)	1(1.9)	3(2.4)	
**By using teleconsultation, are you convinced that you can explain to the patient his diagnosis?**	Totally agree	Observed(%)	3(5.7)	1(4.8)	5(9.6)	9(7.1)	0.443
	Agree	Observed(%)	15(28.3)	3(14.3)	18(34.6)	36(28.6)	
	Neutral	Observed (%)	10(18.9)	4(19)	8(15.4)	22(17.5)	
	Disagree	Observed(%)	14(26.4)	11(52.4)	13(25)	38(30.2)	
	Strongly disagree	Observed (%)	11(20.8)	2(9.5)	8(15.4)	21(16.7)	
**By using teleconsultation, are you convinced that you can explain to the patient the results of imaging, blood tests or additional tests?**	Totally agree	Observed (%)	2(3.8)	3(14.3)	5(9.6)	10(7.9)	0.238

**Table 2(suite):** survey questions, number and response rates by specialty groups

**By using teleconsultation, are you convinced that you can explain to the patient the results of imaging, blood tests or additional tests?**	Totally agree	Observed (%)	2 (3.8)	3 (14.3)	5 (9.6)	10 (7.9)	0.238
	Agree	Observed (%)	28 (52.8)	6 (28.6)	26 (50)	60 (47.6)	
	Neutral	Observed (%)	6 (11.3)	1(4.8)	5 (9.6)	12 (9.5)	
	Disagree	Observed (%)	11(20.8)	10(47.6)	13(25)	34(27)	
	Strongly disagree	Observed (%)	6 (11.3)	1 (4.8)	3 (5.8)	10 (7.9)	
**Do you feel that the inability to perform a physical examination directly limits your ability to correctly diagnose the patient and/or generate the most appropriate treatment plan?**	Totally agree	Observed (%)	18(34)	9 (42.9)	30 (57.7)	57 (45.2)	0.069
	Agree	Observed (%)	21 (39.6)	10 (47.6)	16(30.8)	47(37.3)	
	Neutral	Observed(%)	6(11.3)	1(4.8)	6 (11.5)	13 (10.3)	
	Disagree	Observed (%)	5 (9.4)	1 (4.8)	0 (0.0)	6 (4.8)	
	Strongly disagree	Observed (%)	3(5.7)	0 (0.0)	0 (0.0)	3(2.4)	
**By using teleconsultation, are you able to explain to the patient the appropriate treatment for his state of health?**	Totally agree	Observed (%)	3(5.7)	2(9.5)	5(9.6)	10(7.9)	χ^2^ 15.9 df 8 p
	Agree	Observed (%)	20(37.7)	4(19.0)	27(51.9)	51(40.5)	0.043
	Neutral	Observed (%)	13(24.5)	6(28.6)	13(25.0)	32(25.4)	
	Disagree	Observed (%)	12(22.6)	9(42.9)	5(9.6)	26(20.6)	
	Strongly disagree	Observed (%)	5(9.4)	0 (0.0)	2(3.8)	7(5.6)	
**By using teleconsultation, can you adequately answer the patient's questions?**	Totally agree	Observed (%)	7(13.2)	4(19.0)	6(11.5)	17(13.5)	χ^2^ 15.5 df 8
	Agree	Observed (%)	30(56.6)	4(19.0)	24(46.2)	58(46.0)	p
	Neutral	Observed (%)	7(13.2)	6(28.6)	11(21.2)	24(19.0)	0.050
	Disagree	Observed (%)	5(9.4)	7(33.3)	10(19.2)	22(17.5)	
	Strongly disagree	Observed(%)	4(7.5)	0 (0.0)	1(1.9)	5(4.0)	
**Do you think that the lack of legal texts on the reimbursement and billing of teleconsultation is a handicap for its development?**	Totally agree	Observed (%)	22(41.5)	8(38.1)	16(30.8)	46(36.5)	0.541
	Agree	Observed (%)	21(39.6)	7(33.3)	20(38.5)	48(38.1)	
	Neutral	Observed(%)	5(9.4)	4(19.0)	13(25.0)	22(17.5)	
	Disagree	Observed (%)	4(7.5)	2(9.5)	3(5.8)	9(7.1)	
	Strongly disagree	Observed (%)	1(1.9)	0 (0.0)	0 (0.0)	1(0.8)	
**During the COVID-19 pandemic, do you think patients were absent the opportunity for face-to-**	Totally agree	Observed (%)	18(34.0)	10(47.6)	8(15.4)%	36(28.6)	0.007
	Agree	Observed (%)	27(50.9)	8(38.1)	25(48.1)	60(47.6)	
	Neutral	Observed (%)	6(11.3)	0 (0.0)	12(23.1)	18(14.3)	
	Disagree	Observed (%)	2(3.8)	3(14.3)	7(9.5)	12(13.5)	
**face medical visits?**	Strongly disagree	Observed (%)	0 (0.0)	0 (0.0)	0 (0.0)	0(0.0)	
**After the covid-19 pandemic, are you ready to continue conducting teleconsultations?**	Yes		30(56.6)	8(40.0)	21(40.4)	59(47.2)	0.368

**Tools used during teleconsultation**: prior to the COVID-19 pandemic, teleconsultation was regularly used by ROs, CSs and MOs with a percentage of 23.1%, 26.9% and 61.9% respectively (p=0.035). During the pandemic, only 59.5% of its practitioner resorted to teleconsultation with no significant difference between the three groups (p=0.294). It should also be noted that there is no significant difference in terms of age. Indeed, practitioners aged 45 and over-used teleconsultation more often than younger doctors (25.0% vs. 19.4%) (p=0.406). For teleconsultation tools, 97.5% of participants reported that their institutions do not have a dedicated system and that more than 51.2% used their own means. Indeed, WhatsApp was used as the main means of communication by 42.3% of ROs and 49.0% of CSs while the audio tool was used by 57.9% of MOs. The audiovisual communication tool, WhatsApp, was preferred by doctors over 45 years of age (p=0.339).

**Cancerologists´ satisfaction during teleconsultation**: the comparison of the satisfaction rate between the mode of audio and video communication did not show any significant difference except for their ability to explain to the patient the appropriate treatment for their health condition (p=0.0005). In addition, 42.9% of participants considered that a complete teleconsultation was impersonal (p=0.323). As for their ability to explain the medical diagnosis to a patient, there was no significant difference between the 3 groups (p=0.443). Similarly, 56.5% said they could explain to the patient the results of imaging, blood tests or additional tests without significant differences (p=0.238). On the other hand, 82.5% (p=.069) agreed (ROs: 39.6%, MOs: 47.6%, CSs: 30.8%) or strongly agreed (ROs: 34.0%, MOs: 42.9%, CSs: 57.7%) that not performing a physical examination directly limits their ability to correctly diagnose the patient and generate the most appropriate treatment plan. Nevertheless, ROs and CSs can adequately answer their patients' questions with a significant difference compared to MOs (p=0.05). When it came to their ability to explain appropriate treatment to the patient via teleconsultation, the difference between ROs, CSs and MOs was significant (p= 0.043). Indeed, 42.9% of MOs confirmed it against 32% for ROs and 14.3% for CSs. Health restrictions have hindered patients from heading to oncology centers. Indeed, ROs, MOs and CSs confirmed this with a percentage of 84.9%, 85.7% and 63.5% respectively (p=0.007).

**Teleconsultation practices after COVID-19 pandemic**: the lack of legal texts on the reimbursement and billing of teleconsultation constitutes a handicap for its development according to the practitioners. Indeed, 75.5% of practitioners under 45 years of age confirmed this, compared with 71.5% of those over 45 years of age, with a significant difference (p=0.014). Finally, 47.2% of the participants confirmed that they would be willing to use teleconsultation after the COVID-19 pandemic and 18.4% of the practitioners reported that they would only practice it if it would be remunerated without significant differences in relation to age or specialty.

## Discussion

With this survey, we evaluated the perception and experience of Moroccan oncologists towards the use of teleconsultation during the COVID-19 pandemic. In this study, more than half of the practitioners were practicing in university hospitals. Indeed, this rate could be explained by the fact that in Morocco half of the cancerologists work in UHC.

**Tools used during teleconsultation**: prior to the COVID-19 pandemic, 80% of our survey participants reported offering teleconsultation to their patients. It should also be noted that 90% of MOs used teleconsultation compared to 79% for ROs and CSs. This slight difference by specialty could be due to the more technical side of the last two disciplines which requires the physical presence of the patient for treatment. The rate of use of teleconsultation by our practitioners is much higher than the 5% reported in France and 16.8% has been found in the USA [24-26]. The difference between the value found and those in the literature could be due to the remoteness of cancer patients from health care facilities, the lack of involvement of family physicians in their care and also to the consideration of their socio-economic difficulties by offering them an unpaid service. During the pandemic, 65% of our participants felt it was necessary to use teleconsultation. In contrast, only 45% of Canadian cancerologists, regardless of specialty, preferred teleconsultation to face-to-face consultation [27]. Furthermore, it should be noted that there was no significant difference between specialties. For the transition to teleconsultation, in the USA 79.5% of ROs were more convinced vs. 70% in our series. A difference between MOs and ROs was reported in an Italian satisfaction survey of patients and cancerologist physicians [28]. Indeed, 67% of ROs were totally convinced that teleconsultation should be continued after the end of the pandemic vs. 47% of MOs. Concerning the declaration of never using it afterwards, they were 17%, 16% and 1% respectively for ROs, MOs and CSs [28]. Our survey also revealed that few cancerology facilities have a dedicated platform for audiovisual teleconsultation and that most practitioners used their own means of communication. On the other hand, the majority of our clinicians have adopted the easiest and the most accessible modes of communication for patients (audio communication by phone, SMS and through social networks (WhatsApp, Messenger, etc). This finding has been noted by several previous studies. Indeed, the audio mode alone was adopted by 19.2% of the ROs despite the fact that the majority of the American cancerology centers (93.2%) are equipped with dedicated platforms guaranteeing confidentiality and also by 71% of ROs in a similar Italian study [26,28]. Moreover, the mode of consultation by telephone has been adopted according to the practitioner's specialty. Indeed, ROs, MOs and other specialties used it with a rate of 86.8%, 78.9% and 48.6% respectively (P < 0.001) [28]. In contrast, no physician reported using audio-visual consultations [28].

**Cancerologists´ satisfaction during teleconsultation**: regarding the ability to explain to the patient their medical diagnosis, imaging results, blood tests or additional tests, more than 76% of the participants in the study of Chhabra *et al*. have estimated they could do so [25]. In contrast, only half of the physicians in our series who stated this with significant differences between the 3 groups in favor of the ROs. In fact, the difference between ROs and MOs could be explained by the fact that ROs are in daily contact with equipment and technologies that are constantly evolving. The age of the practitioner could also be one of the factors that intervene to facilitate communication with the patient. Indeed, in the study by Gondal *et al*. the patient satisfaction rate was correlated with the age of the practitioners (≥ 65 years) with a p-value of 0.02 [29]. The result found in our series confirms this because the OMs who were relatively younger than the other two groups confirmed having more difficulties during the teleconsultation. On the other hand, 42% of our clinicians showed that completing teleconsultations was impersonal. Indeed, this rate is slightly lower than reported by Chhabra *et al*. (48%) [25]. Furthermore, almost 80% of our participants reported that not performing a direct physical examination limits their ability to correctly diagnose the patient and/or generate the most appropriate treatment plan. This finding has been reported by several previous studies and also by learned societies [10,30-33]. To partially overcome this handicap, several alternatives would be used namely connected objects (e.g., digital stethoscope) or mobile applications (e.g., blood pressure (e.g., blood pressure monitor, heart rate monitor etc.) or the simultaneous presence of a caregiver next to the patient or the integration of other telemedicine tools (automatic conversational agents ("chatbot”), wearable sensors and mobile health applications) [24,31,33]. Thus, ESMO, following a systematic review by the Multinational Association of Supportive Care in Cancer (MASCC) Survival Study Group, recommended that teleconsultation reports benefits in the management of the psychosocial and physical effects of cancer. It also advocated for more studies to evaluate the use of teleconsultation in the prevention and monitoring of recurrence and new cancers [30,34,35].

**Teleconsultation practices after COVID-19 pandemic**: finally, to date, the absence of a legal framework on the reimbursement of teleconsultation acts in Morocco has pushed 19% of our doctors not to do it without significant difference between the three groups. This is consistent with what was reported in the study by Hamilton *et al*. [27]. Indeed, the lack of technical equipment and especially the lack of reimbursement were obstacles to the development of telemedicine in Australia [27]. Despite these disabilities, many of the physicians in our sample have stated that they will continue to use teleconsultation after the COVID-19 pandemic. This same finding has been reported by similar studies that have also recommended the introduction of teleconsultation in current practices after the COVID-19 pandemic [18,25].

## Conclusion

We believe that the organizational aspects of cancer care continue to be influenced by the consequences of COVID-19. The results obtained from this survey highlight that cancer physicians have been satisfied with their experiences with conducting teleconsultation up to now and have agreed that it will likely be part of their long-term practice. Further studies will be needed to assess patient satisfaction and perception of teleconsultation. Finally, an effort remains to be made in Morocco to equip cancer centers with the technical means guaranteeing the security and confidentiality of teleconsultation.

### What is known about this topic

- *The practices of Moroccan cancerologists in terms of teleconsultation have not yet been explored*
- *The procedures for teleconsultation are not yet documented*.

### What this study adds

- *The majority of cancerologists confirmed the use of teleconsultation before and during the COVID-19 pandemic;*
- *Cancerologists have been satisfied with their experiences with conducting teleconsultation;*
- *Almost half of cancerologists confirmed that they would be ready to use teleconsultation after COVID-19*.
